# Mammary tumors alter the fecal bacteriome and permit enteric bacterial translocation

**DOI:** 10.1186/s12885-022-09274-0

**Published:** 2022-03-05

**Authors:** Brett R. Loman, Kathryn L. G. Russart, Corena V. Grant, Alexis J. Lynch, Michael T. Bailey, Leah M. Pyter

**Affiliations:** 1 Center for Microbial Pathogensis, Abigail Wexner Research Institute at Nationwide Children’s Hospital, Columbus, OH USA; 2 Department of Psychiatry and Behavioral Health & Neuroscience, Institute for Behavioral Medicine Research, 460 Medical Center Drive, Columbus, OH 43210 USA; 3 Comprehensive Cancer Center, The Ohio State University, Columbus, OH USA; 4 Department of Pediatrics, Ohio State University College of Medicine, Columbus, OH USA; 5 Oral and Gastrointestinal Microbiology Research Affinity Group, Abigail Wexner Research Institute at Nationwide Children’s Hospital, Columbus, OH USA

**Keywords:** Cancer, Intestinal barrier, Microbiome, Tumor resection, Inflammation

## Abstract

**Background:**

Cancer patients experience gastrointestinal and behavioral symptoms, and are at increased risk of systemic infection and inflammation. These conditions are a major source of morbidity and decreased quality of life prior to cancer treatment, but poorly defined etiologies impede successful treatment. The gastrointestinal microbiota shape inflammation, influence cancer progression and treatment, and colonize tumors. However, research has not directly determined if peripheral tumors influence the microbiome and intestinal physiology, thus influencing gastrointestinal and behavioral symptoms. Therefore, the purpose of this study was to examine consequences of orthotopic, syngeneic mammary tumor implantation, growth, and resection on fecal bacteriome composition and intestinal barrier function in relation to systemic inflammation and enteric bacterial translocation in mice.

**Methods:**

Female mice were randomized to 3 experimental groups: sham surgical control, tumor recipients, and tumor recipients later receiving tumor-resection. Mice were sacrificed three weeks after tumor implantation or resection for collection of stool, colon, spleen, and brain tissue and analysis.

**Results:**

Tumor-bearing mice exhibited several markers of colonic barrier disruption, including dampened expression of tight junction proteins (*Cldn1* and *Ocln*) and elevated circulating lipopolysaccharide binding protein (LBP). Compromised colonic barrier integrity was associated with altered fecal bacterial profiles in tumor-mice, including lower relative abundance of *Lactobacillus*, but higher *Bacteroides*. Consistent with colonic barrier disruption and altered microbiomes, tumor-mice displayed markers of systemic inflammation including splenomegaly, higher splenic bacterial load, and elevated splenic and brain pro-inflammatory cytokines. Several  bacteria cultured from spleens had 16S rRNA gene amplicons matching those in fecal samples, suggesting they were of intestinal origin. Fecal *Lactobacillus* was highly-interrelated to physiological parameters disrupted by tumors via correlation network analysis. Tumor resection ameliorated circulating LBP, splenomegaly, and splenic cytokines, but not other parameters associated with loss of colonic barrier integrity and bacterial translocation.

**Conclusions:**

Orthotopic mammary tumors alter the microbiome, reduce intestinal barrier function, increase translocation of enteric bacteria, and alter systemic inflammation. This provides insight into how tumors commence gastrointestinal and behavioral symptoms prior to treatment, and identify targets for future therapeutics, such as probiotic *Lactobacillus* supplementation.

**Supplementary Information:**

The online version contains supplementary material available at 10.1186/s12885-022-09274-0.

## Background

Cancer patients commonly experience gastrointestinal (GI) and/or behavioral symptoms including nausea, diarrhea, anxiety, and depression [1–4]. Although GI symptoms are not surprising in patients with tumors of the abdominal cavity or after the initiation of cancer treatment, these symptoms are often evident even prior to diagnosis and are not restricted to abdominal cancers [2, 5]. Moreover, these symptoms can persist well into survivorhood. For women with breast cancer, up to one-fourth experience GI symptoms and one fifth experience cognitive symptoms prior to treatment [1, 6]. While inflammation from direct tissue damage is implicated in causing these symptoms following treatment, mechanistic insight into the development of these symptoms prior to treatment remains sparse [3]. Gastrointestinal and cognitive symptoms are major sources of morbidity and distress for cancer patients, yet treatment options remain limited due to complex and poorly defined etiologies [7, 8]. Therefore, research detailing the underlying pathophysiology of these symptoms will identify targets for development of effective therapeutic options.

Multiple interconnected physiological systems can influence the development of these symptoms, such as the microbiota-gut-brain axis. No studies currently address the contribution of the enteric microbiota to tumor-related GI and behavioral symptoms, but thesese microorganisms likely participate given their abilities to influence development and treatment of cancer, [9, 10] shape the immune system, [11] modulate behavior via the gut-brain axis, [12] and potentiate GI symptoms [13, 14]. For example, changes in the intestinal microbiota are implicated in initiation and progression of colon polyps and tumors, as well as cancer-associated GI dysfunction and cachexia [15, 16]. The fecal microbiome is altered in patients with tumors both proximal and distal to the GI tract prior to treatment, but previous studies are unable to assign causality to these changes (e.g. whether the tumor is changing the microbiome or vice versa) [17–19]. Therefore, existing research has yet to directly establish whether tumors distal to the GI tract are capable of altering the intestinal microbiome.

Systemic bacterial infection arises in cancer patients due to immune deficiencies, exacerbating systemic inflammation [20]. Although the source of these microbes has not been confirmed, bacterial taxa typically commensal to the intestine have been detected in the bloodstream of patients with cancer [21, 22]. Importantly, such systemic bacterial infections drive inflammation, increasing the propensity of short and long-term symptoms [23]. Recently, the human tumor microbiome was examined across a myriad of tumor types.^17^ Intriguingly, mammary tumors possess the highest diversity of tumor-associated bacteria, including those common to the intestinal tract such as Lactobacillales, Bifidobacteriales, and Bacteroidales [22]. However, the source, viability, and physiological consequences of bacteria colonizing tumors and systemic body sites remains unclear.

Therefore, the purpose of this study is to examine the consequences of tumor growth and resection on fecal bacteriome composition and colonic barrier function in relation to systemic inflammation and enteric bacterial translocation in mice. In an orthotopic and syngeneic mammary tumor model, tumor-bearing mice display decreased colonic barrier function, altered bacterial metataxonomic signatures, and altered splenic and brain pro-inflammatory cytokine production in conjunction with increased splenic burden of commensal enteric bacteria, many of which persist even after tumor resection. We conclude that peripheral tumors likely instigate gastrointestinal and behavioral symptoms prior to and independent of treatment (in opposition to the belief that cancer treatment solely instigates these side effects) through perturbed colonic microbial populations and barrier disruption, and treatments that maintain positive host-microbial interactions in the colon represent promising therapeutic options.

## Methods

### Animals

**Fig. 1:**
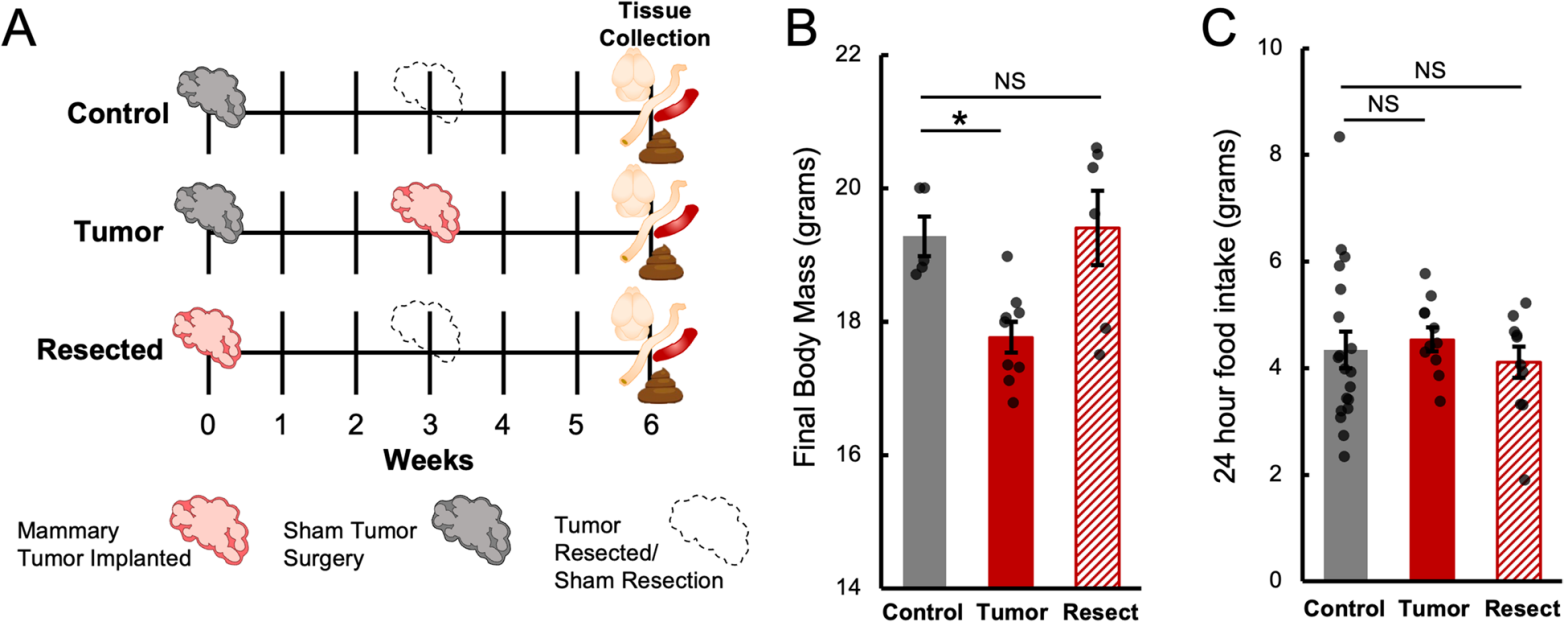
Mammary tumors reduce body mass without diminishing food intake. Orthotopic and syngeneic breast tumors are implanted and resected as in (**A**) prior to tissue collection. Tumor-bearing mice lose non-tumor body mass, but this effect is attenuated by Resection (**B**). Weight loss in this model is not due to reduced food intake (**C**). * = *p* < 0.05, NS=Not Significant, by ANOVA and post-hoc LSD

**Fig. 2:**
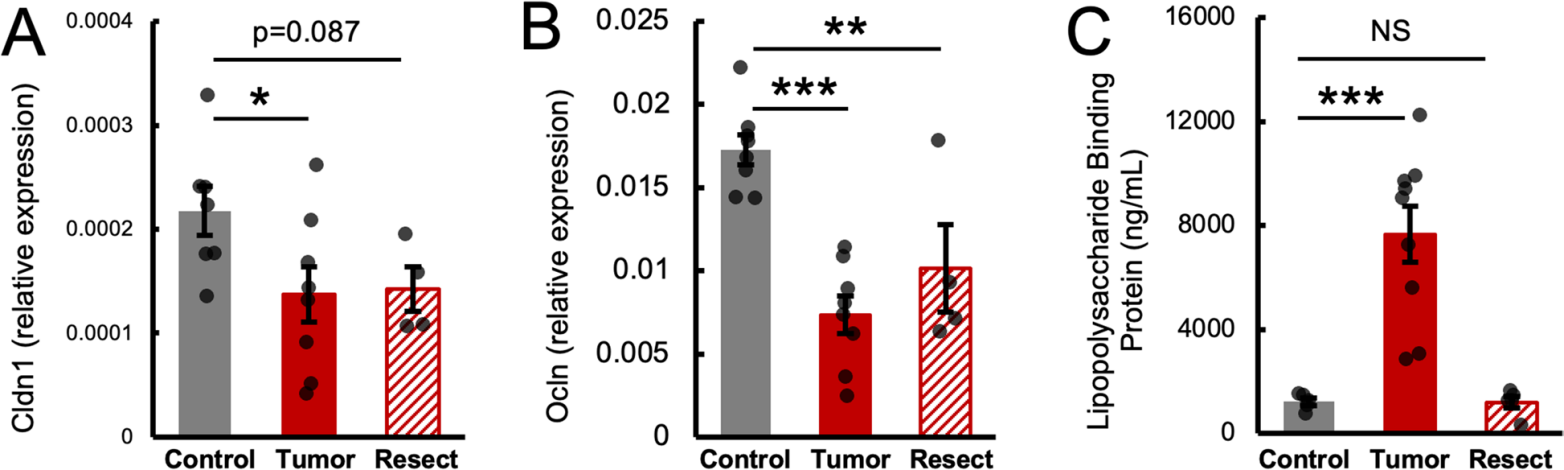
Mammary tumors impair markers of colonic barrier integrity. Tumor-bearing mice have decreased colonic expression of tight junction proteins *Claudin 1* (*Cldn1*, **A**) and *Occludin* (*Ocln*) (**B**). Resected mice have reduced *Ocln* expression and tend to have reduced expression of *Cldn1*. Tumor mice also have increased circulating lipopolysaccharide binding protein (**C**), a marker of bacterial translocation. * = *p* < 0.05, ** = *p* < 0.01, *** = *p* < 0.0001, NS=Not Significant, by ANOVA and post-hoc LSD

**Fig. 3:**
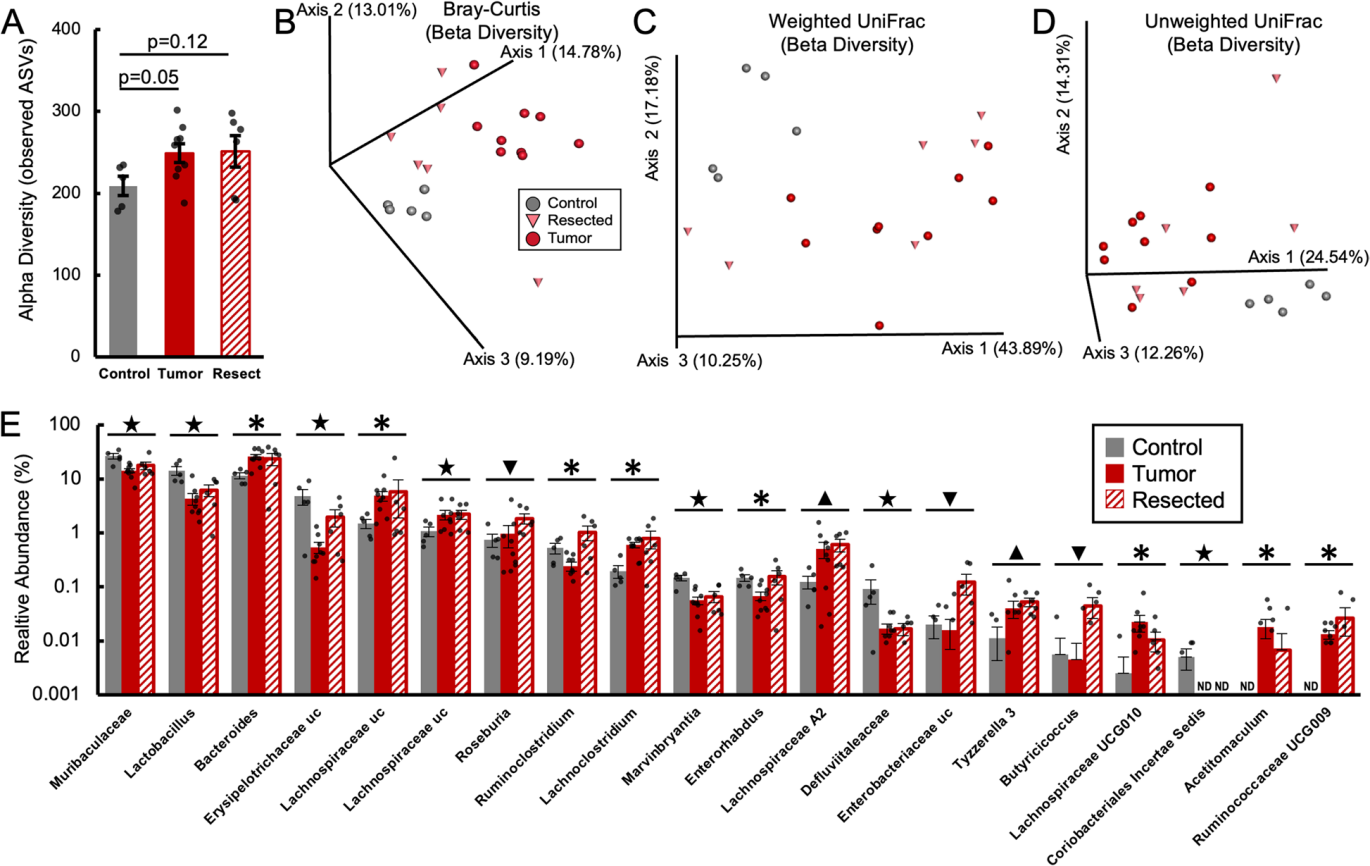
Mammary tumors alter the fecal bacteriome, even following resection. 16S rRNA gene amplicon sequencing reveals that Tumor and Resected mice tend to have higher fecal bacterial diversity as measured by observed amplicon sequence variants (ASVs) relative to Control mice (**A**). Tumor, Resected, and Control mice have distinct fecal bacterial populations as measured by: Bray-Curtis (*p* = 0.001) (**B**), Weighted UniFrac (*p* = 0.004) (**C**), and Unweighted UniFrac distances (*p =* 0.001) (**D**). Control, Tumor, and Resected mice also host multiple different relative abundances of bacterial genera (**D**).  ★= Tumor and Resected different from Control, *= Tumor different from Control, ▼= Resected different from Tumor and Control, Triangle (up)▲ = Resected different from Control

**Fig. 4:**
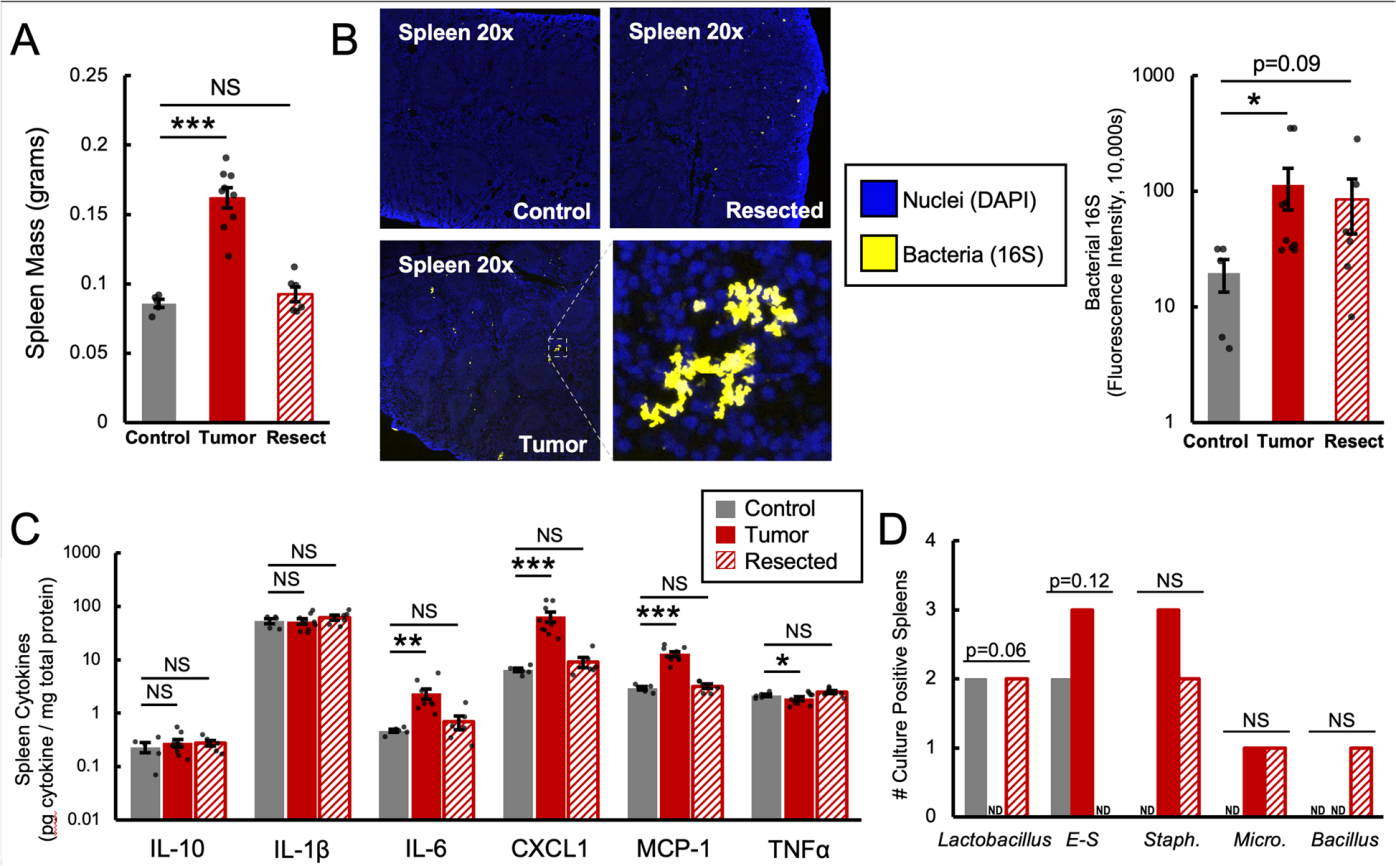
Mammary tumors increase splenic bacterial burden and alter splenic cytokines. Tumor mice exhibit splenomegaly that is attenuated in Resected (**A**). Tumor mice also have increased splenic bacterial burden as detected by FISH (**B**). Tumor mice also have altered splenic cytokine profiles (**C**). Control, Tumor, and Resected mice do not differ in the number of animals with culture-positive spleens (60, 66.7, and 66.7% respectively,chi-square likelihood ratio *p* = 0.96), but Tumor mice tend to have less *Lactobacillus*-positive spleens (**D**). * = *p* < 0.05, ** = *p* < 0.01, *** = *p* < 0.0001, NS=Not Significant, ND=Not Detected, by ANOVA and post-hoc LSD

**Fig. 5:**
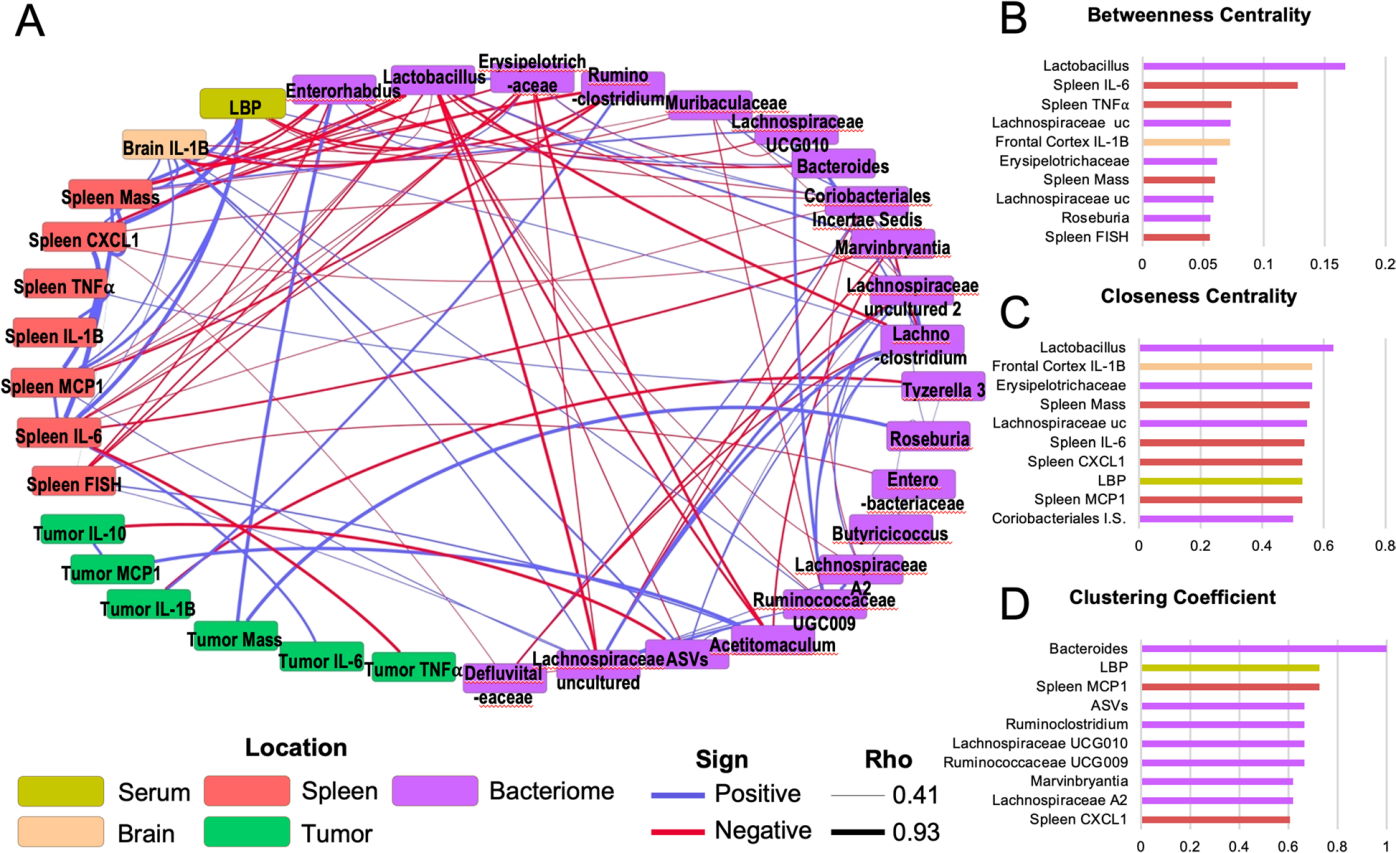
High-abundance bacterial genera are greatly related to other tumor-induced physiological changes. Parameters that were significantly different by tumor status are highly interrelated as shown by correlation network analysis (**A**). *Lactobacillus* seems highly related to tumor-induced changes in that this genera has the highest betweenness centrality (**B**) and closeness centrality (**C**) (measures of high inter-connectively) among all other included parameters. *Bacteroides* (another high-abundance genus) also clusters perfectly with all other related parameters (clustering coefficient of 1), and LBP-related parameters are almost all also related to one another (clustering coefficient 0.73) (**D**) ND = Not Detected, E-S = *Escherichia-Shigella, *Staph. = Staphylococcus, Micro. = micrococcus.

All animal experiments were approved by The Ohio State University Institutional Animal Care and Use Committees and carried out in accordance with the National Institutes of Health Guide for the Care and Use of Laboratory Animals. Anesthesia and euthanasia procedures followed American Veterinary Medical Association guidelines. Two cohorts of nulliparous female 7- to 8-week old Balb/c mice (purchased from Charles River, Wilmington, MA) were housed 5/cage and acclimated to the temperature-controlled (22 ± 1 °C) vivarium with a 14:10 light:dark cycle (lights off at 015:00 h). Rodent chow (Teklad 7912) and water were available ad libitum throughout the study and cotton nestlets and plastic huts were provided for nesting. After 2 weeks of acclimation, mice were pseudorandomly assigned to 3 experimental groups to equalize initial body mass amongst groups: sham surgical control (Control), tumor recipients (Tumor), and tumor recipients that later received tumor resection (Resected) (Fig. 1A). Each animal served as an experimental unit and none of the data collected was excluded. Researchers were not blinded to treatment groups, as monitoring tumor mass was part of humane animal study endpoints. Sample size in each experiment was calculated to ensure 80% power with 0.05 type-I error for the primary analysis derived from our previous studies. Cohort N_1_ was utilized for food intake and colon barrier function data (Figs. 1A, C and 2, Control *n* = 19, Tumor *n* = 11, Resected *n =* 11), while cohort N_2_ was utilized for all other data presented (Figs. 3, 4 and 5, Control *n* = 5, Tumor *n* = 9, Resected *n* = 6). 24-h food intake was measured between days 18–19 post tumor induction (Tumor, Control) or tumor resection (Resected). Body mass was taken just prior to animal sacrifice.

### Tumor cells

Non-metastatic murine 67NR mammary tumor cell line, which originated from a spontaneous mammary adenocarcinoma in a Balb/c mouse [24] was generously provided by Drs. Fred Miller and Lisa Polin at Karmanos Cancer Institute (Detroit, MI USA). Cells were cultured in Dulbecco’s Modified Eagle Media with 10% FBS, 2 mM _L_-glutamine, 1 mM nonessential amino acids, and 100U/mL Pennicillin-Streptomycin at 37 °C and 5% CO_2_.

### Tumor induction

To induce mammary tumors, 5 × 10^6^ tumor cells (embedded in Matrigel, phenol red-free, BD Biosciences) were surgically inoculated into the 4th mammary fat pad of mice anesthetized with isoflurane vapors as previously described [25]. Two unique strengths of this mammary tumor model are: 1) the tumors stem from a syngeneic murine cell line, allowing for completely immunocompetent mice, and 2) the surgical intra-mammary fat pad tumor cell inoculation makes the tumor orthotopic. Most mouse models of cancer rely on immunodeficient mice and heterotopic tumors, which greatly limit the translational relevance. Tumors were induced in Resected mice approximately 2 weeks prior to the Tumor mice to allow time for tumor resection and healing prior to tissue collection (see Fig. 1A). Body mass and tumor dimensions (palpable 7–9 d post-induction) were measured 2x/week, and control mice were similarly handled.

### Tumor resection

Tumors of the Resected mice were surgically removed using a modified radical mastectomy procedure as previously described [25]. Mice were anesthetized with isoflurane, and tumors were surgically removed along with mammary tissue, fat, and lymph nodes. Buprenorphine (0.05 mg/kg; s.c) was administered immediately after surgery and every 6–12 h post-surgery for 72 h. Sham surgeries were conducted in the control and tumor groups so that each mouse received 2 surgeries to control for effects of anesthesia and wound healing (see Fig. 1A).

### Gene expression

Colons were collected and frozen on dry ice for RT-qPCR. Brains were removed, and the frontal cortex -a brain region that regulates mood and cognition- was collected via fresh dissection prior to freezing on dry ice. RNA was isolated from the distal colon and frontal cortex with the RNeasy Mini Kit (Qiagen) according to the manufacturer’s protocol. RNA concentration and purity was measured using a NanoDrop™ Spectrophotometer (Thermo Fisher Scientific). Equal amounts of RNA for each sample were reverse transcribed using the qScript® cDNA SuperMix kit (Quantabio) according to manufacturer’s recommendations. Gene expression was measured using TaqMan™ Fast Advanced Master Mix, TaqManTM predesigned gene expression primers/probes (Thermo Fisher Scientific) and amplified using a QuantStudio™ 5 Real-Time PCR System (Thermo Fisher Scientific). Expression was quantified using the ΔCt method with *Gapdh* as the endogenous control.

### LBP immunoassay

Mice were euthanized by rapid decapitation and trunk blood was collected using a heparinized Natelson tube. Plasma was separated from whole blood by centrifugation and stored at − 80 °C. Lipopolysaccharide binding protein (LBP) concentration was quantified in plasma using the LBP Mouse ELISA kit (HycultBiotech) according to the manufacturer’s protocol.

### Immunohistochemistry

The distal colon was removed, washed with PBS, and fixed in 10% formalin for 24 h. Distal colons were sectioned and stained by the Comparative Pathology and Mouse Phenotyping Shared Resource at The Ohio State University. Heat-induced epitope retrieval was performed in a vegetable steamer for 20 min in pH 6 citrate buffer, and anti-F4/80 antibody (clone MCA497, BioRad Laboratories, Hercules, CA) was utilized as a marker of monocytes and macrophages to assess infiltration into colon tissue.

### Fecal Bacteriome sequencing

Two days prior to tissue collection, fecal samples were collected from each mouse and flash frozen on dry ice. Stool samples were sent to The Environmental Sample Preparation and Sequencing Facility at Argonne National Laboratory for DNA extraction, library preparation, and high-throughput sequencing. Paired-end (250 nt forward and reverse) sequences of the V4 hypervariable region of the 16S rRNA gene (515F-806R) were generated on the Illumina MiSeq. Quantitative Insights into Microbial Ecology (QIIME) 2.0 [26] was utilized for amplicon processing, quality control with DADA2, and downstream taxonomic assignment using the SILVAv132 database [27]. Sequencing of these samples initially resulted in 1,834,408 paired-end sequences (median = 93,912; maximum = 118,636; minimum = 46,468). After quality control, 1,103,728 high-quality sequences remained (median = 55,832; maximum = 71,371; minimum = 32,520). Samples were rarefied to 32,520 sequences for downstream analyses and no samples were excluded.

### Fluorescence in-situ hybridization (FISH)

Spleen and Tumor tissues were fixed in methacarn (60% methanol, 30% chloroform, 10% acetic acid) overnight, embedded in paraffin, and sectioned at 4 μm on positively charged microscope slides. Sections were dewaxed by heating on a heat block at 60 °C for 10 min, two subsequent incubations in xylene substitute at 35 °C and room temperature respectively for 10 min each, and a final incubation in 100% ethanol for 5 min. Then sections were incubated in hybridization buffer (0.9 M NaCl, 0.02 M Tris-HCl, 20% formamide, 10% SDS) for 10 min at 50 °C in a humidified slide chamber in a hybridization chamber, dried and outlined with a PAP pen, then incubated with 0.5 μg of probe EUB (/5Cy3/GCTGCCTCCCGTAGGAGT/3Cy3Sp/) under a cover slip in hybridization buffer in a humidified slide chamber in a hybridization chamber overnight. The next day, sections were washed with washing buffer (0.9 M NaCl, 0.02 M Tris-HCl) twice for 10 min, incubated with DAPI for 15 min, and washed once with PBS for 5 min. Finally, sections were processed for autofluorescence reduction and mounting with the Vector TrueVIEW Autofluorescence Quenching Kit (Vector Laboratories, Burlingame, CA) according to manufacturer instructions.

### Cytokine immunoassay

Spleen and tumor fragments were lysed via sonication (20 s, 25% amplitude) in 150–300 μL of 50 mM Tris-base buffer (0.2 mM PMSF, 100 mM amino-n-caproic acid, 10 mM EDTA, 5 mM benzamidine) plus one cOmplete mini protease inhibitor cocktail tablet per 10 mL of buffer (Millipore Sigma). Samples were centrifuged at 14,000 RPM at 4 °C for 10 min and the supernatant retained. A standard Bradford assay was used to determine the protein concentration of each sample. The concentration of IL-10, IL-1β, IL-6, CXCL1, MCP-1, and TNFα were measured using a multi-plex cytokine array (U-Plex, MesoScale Discovery) according to manufacture protocol. Briefly, 125 μg of tumor protein or 50 μg of spleen protein was incubated overnight at 4 °C while shaking in the antibody-coated, U-PLEX Biomarker Group 1 (ms) Assays, SECTOR plate. The plate was washed, incubated with detection antibody solution, washed, and read immediately following the addition of Read Buffer. The plate was read on a MESO QuickPlex SQ 120 Instrument. The signal CV for each cytokine/chemokine were as follows: IL-1β and TNFα ≤5%, CXCL1 and MCP-1 ≤ 10%, and IL-6 ≤ 15%.

### Bacterial culture and identification

Flash-frozen spleen tissue was brought to room temperature in 1 mL PBS, pulverized with autoclaved mortar and pestle, and plated on autoclaved BBL Schaedler agar (BD Biosciences, San Jose, CA). Plates were incubated for 5 days in a humidified incubator at 37 °C with 5% CO_2_. Individual colonies were then transferred to 5 mL BBL Schaedler broth (BD Biosciences, San Jose, CA) for 5 days under the same incubation conditions. After 5 days, cultures were centrifuged at 5000 g for 10 min, the supernatant was removed, and DNA was isolated with the QIAamp Fast DNA Stool Mini Kit (Qiagen, Hilden, Germany) according to manufacturer instructions.

DNA was quantified using the Qubit high-sensitivity dsDNA quantification assay (ThermoFisher Scientific, Waltham, MA) according to manufacturer instructions, amplified via PCR to produce amplicons of the V3-V5 hypervariable region of the 16S gene (357F-926Rb) [28], cleaned up with DNA Clean and Concentrator-25 (ZYMO Research, Irvine, CA), and sequenced by Eurofins Genomics (Louisville, KY).

### Correlation network analysis

All data that were significantly different in either tumor or resected groups relative to control as well as tumor cytokine concentrations were tested for all potential Spearman correlations. Correlations that were significant at *p* < 0.05 and rho> 0.4 were plotted in a correlation network for analysis via Cytoscape.

### Statistical analyses

Results of body and spleen mass, food intake, gene expression, LBP ELISA, F4/80 IHC, MSD, and spleen FISH were tested for normal distribution and equal variances. Assumptions of parametric testing were met, a one-way ANOVA was used, and followed by multiple t-tests to compare between control, tumor, and tumor resected groups. Bacterial 16S rRNA gene amplicon sequencing data was analyzed via PERMANOVA (diversity), and Wilcoxon (genus-level differential abundances) tests. Spleen culture positivity was tested via Chi-square test. Significance was set at *p* < 0.05.

### Data sharing

The data that support the findings of this study are available from the corresponding author upon reasonable request.

## Results

### Mammary tumors reduce body mass without diminishing food intake

Weight loss and anorexia are common consequences of cancer [15]. In this orthotopic and syngeneic breast tumor model (Fig. 1A), the final body masses of Tumor mice (less the mass of the tumor), but not Resected mice were lower than that of Controls (Fig. 1B). However, this difference was not due to lower food intake, as neither group varied from Control in this model (Fig. 1C).

### Mammary tumors impair markers of colonic barrier integrity

Cancer is associated with increased inflammation and changes in intestinal function [15]. Specifically in this model, mammary tumors produce pro-inflammatory cytokines and tumor implantation increases cytokines in circulation [25, 29]. Proteins such as claudins and occludin are integral to intestinal tight junctions and inflammation-induced changes in their expression are linked to loss of barrier integrity [30]. Tumor-bearing mice had lower expression of tight junction proteins *Claudin-1* (*Cldn1*)(Fig. 2A) and *Occludin* (*Ocln*) (Fig. 2B) in the colon, while Resected mice only had lower expression of *Ocln* (but tended to have lower *Cldn1*) relative to Control mice. Decreased expression of *Cldn1* and *Ocln* can also be related to monocyte infiltration and inflammation, such as in mouse models of colitis [31]. To rule out leukocyte-induced decreases in tight junction protein expression in the colon, immunohistochemistry was performed for F4/80 (a marker of monocytes and macrophages). However, there were no differences in F4/80 staining in Tumor or Resected mice relative to Control (Supplementary Fig. 1). Increased contact with bacteria at the epithelium and higher potential of bacterial translocation is a consequence of decreased intestinal barrier function [32]. Lipopolysaccharide binding protein (LBP) is a circulating marker of this increased epithelial exposure to bacterial lipopolysaccharide and endogenous cytokines [33]. Serum LBP was higher in Tumor mice relative to Control, but attenuated in the Resected group (Fig. 2C), further supporting disruption of the intestinal barrier due to mammary tumor implantation.

### Mammary tumors alter the fecal bacteriome, even following resection

The intestinal microbiome and intestinal physiology are highly responsive to changes in one-another. Intriguingly, bacterial 16S rRNA gene amplicon sequencing revealed altered metataxonomic signatures in Tumor and Resected fecal samples relative to Control. In terms of alpha diversity, both Tumor and Resected animals tended to have higher total observed amplicon sequencing variants (ASVs, a measure of bacterial richness) (Fig. 3A), but there were no differences in Faith’s Phylogenetic Diversity or Pielou’s Evenness (measures of taxonomic diversity and evenness, Wilcoxon *p* = 0.93 and *p* = 0.50 respectively – data not shown). Differences in fecal bacterial populations were more pronounced by measures of beta diversity. All three groups significantly differed from one another by Bray-Curtis, Weighted UniFrac, and Unweighted UniFrac distances (Fig. 3B, C, and D). These differences in beta diversity were underpinned by differences in relative abundances of both high- and low-abundance taxa analyzed at the genus level (Fig. 3E). Of the most abundant taxa represented in the dataset, an unidentified genus in the family *Muribaculaceae* and the genus *Lactobacillus* were less abundant in Tumor and Resected mice relative to Control. Conversely, *Bacteroides* was more abundant in Tumor, but not Resected mice. Furthermore, Tumor and Resected mice shared relatively higher abundances of an uncultured genus in *Lachnospiraceae*, and lower abundances of an uncultured genus in *Erysipelotrichacae*, *Marvinbryantia*, *Defluvitaleaceae*, and *Coriobacteriales Incertae Sedis* relative to Control. Some genera were altered in Tumor samples, but did not persist in Resected samples including: higher abundances of another uncultured genus in *Lachnospiraceae*, *Lachnoclostridium*, *Lachnospiraceae UCG010*, *Acetatomaculum*, and *Ruminococcaceae UCG009*, and lower abundances of *Ruminoclostridium* and *Enterorhabdus* relative to Control. Finally, differences unique to Resected mice included higher abundances of *Roseburia*, *Lachnospiraceae A2*, an uncultured genus from *Enterobacteriaceae*, *Tyzerella 3*, and *Butyricicoccus*.

### Mammary tumors increase splenic bacterial burden and increase systemic inflammation

Higher splenic bacterial burden is common amongst conditions that induce colonic barrier dysfunction and bacterial translocation, including social stress and infectious colitis in mice [34, 35]. Relative to Control, Tumor mice had higher spleen mass (Fig. 4A), higher burden of total splenic bacteria as detected via FISH staining for global bacterial 16S DNA (Fig. 4B), and higher splenic production of the pro-inflammatory cytokines IL-6, CXCL1, and MCP-1, but lower TNFα (Fig. 4C). However, these effects were attenuated in Resected mice. FISH bacterial staining conducted on excised mammary tumors were negative for detectable fluorescence (Supplementary Fig. 2). As a source of inflammatory signals and for correlational analysis, the same cytokines were also quantified from excised tumor tissue (Supplementary Fig. 2). Given that breast cancer in humans is associated with persistent behavioral symptoms [36], expression of immune markers in the brain related to bacterial signaling pathways were also investigated. Inflammation detected in the spleen was mirrored in the brain, specifically by higher expression of *Il-1β*, but without alterations in pathogen-related signaling receptors (Supplementary Table 1).

### Tumor-enhanced splenic bacteria are likely derived from the intestine

In order to discern whether the bacteria quantified by FISH staining in the spleen were viable, spleen tissue was cultured for commensal intestinal microbiota. The number of culture-positive spleens (those that produced at least one colony) was not significantly different between treatment groups, being 60, 66.7, and 66.7% for Control, Tumor, and Resected mice respectively (chi-square likelihood ratio *p* = 0.96). Sequencing of the V4 region of the 16S rRNA gene identified isolates as bacteria from the genera *Lactobacillus, Escherichia-Shigella, Staphylococcus, Micrococcus, and Bacillus* (Fig. 4D). *Lactobacillus* tended to be less likely to be cultured from spleens of Tumor mice, but no other differences were detected. When sequences from the isolates were compared to sequences from fecal microbiome 16S rRNA gene amplicon data, sequences from *Lactobacillus* and *Staphylococcus* had 100% matches, suggesting that the isolates were of intestinal origin (Supplementary Table 2).

### High-abundance fecal bacterial genera are highly related to other tumor-induced physiological changes

Correlation network analysis is helpful to identify which tumor-induced changes are likely influencing one-another. Correlations between tumor-altered parameters were plotted in a network to demonstrate the relationships and patterns between them (Fig. 5A). Relative abundance of fecal *Lactobacillus* was the most-connected parameter with a degree (number of correlations) of 18 out of 35 possible relationships. Notably, the vast majority of these relationships were negative associations with the bacteria, spleen cytokines, spleen FISH staining, brain *Il-1β*, and serum LBP that were all higher in Tumor mice (given that the relative abundance of *Lactobacillus* was lower in Tumor and Resected mice). This was further reflected by network analyses (Fig. 5B and C), where *Lactobacillus* scored highest in both Betweenness Centrality (a measure of network centrality that determines the shortest path [number of connections] between all members of the network and calculates the percentage of paths to which a given member belongs), and Closeness Centrality (another measure of network centrality similar to Betweenness Centrality, but takes into account the length of each connection - in this case the magnitude of the correlation coefficient).

*Bacteroides* -another high-abundance bacteria- scored a perfect 1 for Clustering Coefficient, meaning that 100% of the parameters it was correlated to were also correlated to one-another (Fig. 5D). However this list of connections was rather short (degree = 3), including only LBP, *Enterorhabdus*, and *Erysipelotrichaceae*. Conversely, LBP possessed the second highest Clustering Coefficient but also a high degree (0.73 and 11 out of 35 respectively). LBP was negatively associated with the relative abundances of genera lower in Tumor mice (*Lactobacillus, Enterorhabdus, Erysipelotrichaceae,* and *Ruminoclostridium*), but positively associated with parameters higher in Tumor mice (*Bacteroides,* brain *Il-1β*, spleen mass, and spleen cytokines IL-6, CXCL1, and MCP1). Tumor cytokines were correlated to very few other parameters.

## Discussion

Long-term GI symptoms, behavioral symptoms, and infection are common in cancer patients. While this is evident following cancer treatments including chemotherapy and radiation, these symptoms also occur prior to diagnosis (thus prior to treatment) and can persist for years into survivorhood. This suggests that tumor biology itself plays a role in the development of these symptoms. However, the underlying etiology of these symptoms, and whether the microbiome is involved, have not been systematically studied. This study demonstrates that subcutaneous tumors alter the enteric microbiome and compromise intestinal barrier function - thus allowing for increased translocation of commensal enteric bacteria to other organs - ultimately influencing systemic inflammation that can drive these symptoms. Imperative to advancing cancer treatment, this study also suggests that resection of the tumor does not completely remedy these consequences, and that physiological processes underpinning cancer-related side-effects are present prior to (and likely exacerbated by) treatments such as chemotherapy and radiation.

Based on the few available studies, the fecal microbiome is altered in patients with various types of cancer prior to treatment, but whether these changes are due to the cancer itself or due to other contributing factors is not clear [17–19]. In our study, tumor implantation and growth induced marked differences in fecal bacteriome composition, directly demonstrating that cancer alters the microbiome. These differences were evident both community-wide and at the genus level. Intriguingly, many of these differences persisted after tumor resection, implying long-term consequences in cancer survivors. While not directly investigated in this study, enduring shifts in microbial communities may be related to persistent changes in GI physiology such as altered barrier function, inflammation, or motility. Mice in both Tumor and Resected groups tended to have increased fecal bacterial diversity in terms of total unique bacterial sequences detected. While higher diversity of the intestinal microbiome is generally associated with health, this tendency for increased diversity in tumor-bearing mice may be more reflective of less population control of the microbiome by the host. For example, activity of antimicrobial peptides can reduce gram positive bacteria, and secretion of antibodies by lymphocytes can both enhance and reduce bacterial populations dependent upon their susceptibility [37, 38]. Thus, future investigation into potential differences in these and other bacteria-controlling functions in tumor-bearing animals is of high interest. Differences in alpha diversity were corroborated by differences in beta diversity in this study. Fecal bacterial populations of Resected mice lie somewhere between Control and Tumor mice, supporting the notion that tumor resection alone is not sufficient to restore typical microbial populations. Both Tumor and Resected mice had lower relative abundances of genera in the family *Muribaculaceae* and *Lactobacillus*. *Muribaculaceae* (formerly known as S24–7) is a commensal organism of the mammalian GI tract with the potential for degrading both dietary and host-derived glycans, but little else is currently known of these understudied organisms [39]. *Lactobacillus,* on the other hand, is known for a myriad of health-promoting effects in the gastrointestinal tract including enhanced barrier function, [40] immunomodulation, [41] and influencing intestinal motility [42]. In contrast to those genera, *Bacteroides* was higher in both Tumor and Resected mice. Bacteria belonging to this genus are implicated in colonic inflammation via crypt dysplasia, [43] bile acid metabolism, [44] and disruption of tight junctions (as is also observed in these animals) [45]. Interestingly, the genera *Roseburia* and *Butyricicoccus* were uniquely higher in resected mice. Both of these genera are known for their ability to produce butyrate [46] – a metabolic product of fiber fermentation with extensive roles in colon health [47]. Increased abundance of these bacteria could thus facilitate recovery of the colonic barrier over time, but longer-term investigation into this process is required post-tumor resection. Relative abundances of many genera from the family *Lachnospiraceae* were altered by tumor implantation, although the direction of these differences was inconsistent (e.g. some higher and some lower depending on the genus) as our group has observed in chemotherapy-treated animals, [48] reinforcing the need for increased understanding of the biological processes in which these understudied organisms participate in the gastrointestinal tract. Overall, these shifts in microbial populations may contribute to GI and behavioral symptoms through altered metabolite production and immune interactions that should be investigated in future studies.

**Fig. 6:**
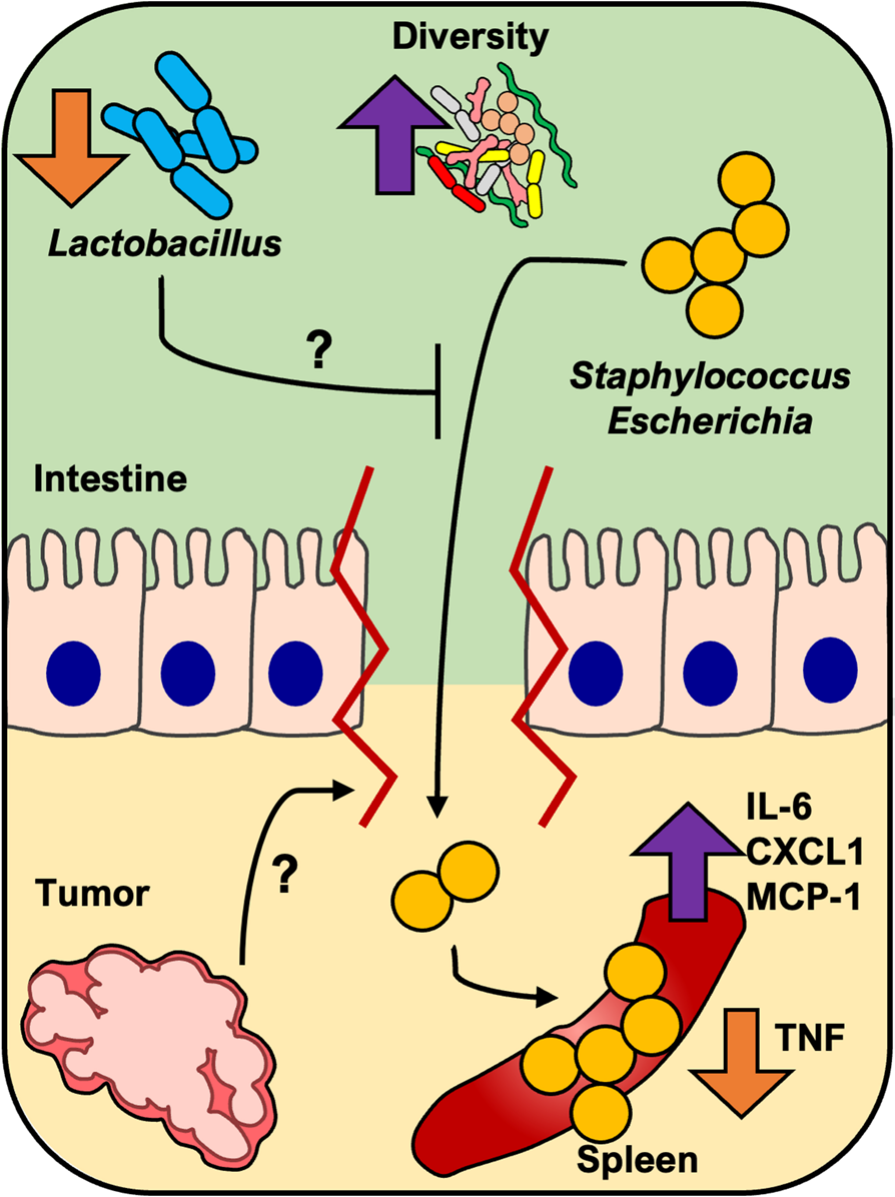
Working paradigm - Tumor-induced intestinal barrier disruption is permissive of enteric bacterial translocation. Tumors induce intestinal barrier disruption and decrease relative abundance of *Lactobacillus* through yet unknown mechanisms. Reduced abundance of *Lactobacillus* and barrier disruption allow higher enteric bacterial translocation to systemic sights including the spleen, where pro-inflammatory cytokine TNFα is reduced. These changes in intestinal physiology and immune function likely contribute to gastrointestinal and behavioral symptoms experienced by patients with cancer

GI and behavioral symptoms can be driven by inflammation, but the source of inflammation underpinning these symptoms is unlikely to be from tumor-secreted factors alone. This study provides evidence that enteric bacterial translocation contributes to systemic inflammation in tumor-bearing animals. In tumor-bearing mice, changes in the fecal bacteriome and intestinal barrier function were associated with both higher bacterial load and altered immune function in the spleen, likely due to enteric bacterial translocation. Through FISH, we identified higher amounts of bacteria that appeared to be in-tact in the spleens of both Tumor and Resected mice. The use of traditional culture techniques demonstrated that these bacteria were indeed viable. Furthermore, we identified these bacteria as normal colonizers of the intestinal tract, supporting our hypothesis that tumor-induced intestinal barrier disruption is permissive of enteric bacterial translocation (Fig. 6). Although spleens from Control mice were also culture-positive, half of these bacteria were identified as *Lactobacillus*, which is associated with multiple intestinal health benefits and are commonly found in the spleens of even healthy mice [34, 35]. By contrast, spleens from Tumor mice tended to be less likely to be culture positive for *Lactobacillus,* suggesting that *Lactobacillus* may exert colonization resistance against other bacteria in the spleen, although that remains undetermined at this time. Additionally, network analyses support the notion that the fecal relative abundance of *Lactobacillus* (lower in both Tumor and Resected groups relative to Control) is important for maintaining normal barrier and immune functions in the intestine and spleen. Specifically, this is illustrated by its negative relationships to circulating LBP, spleen FISH intensity, spleen cytokines, and brain *IL-1β* expression (all suggesting systemic immune activation). Although we did not identify what aspect of tumor implantation and growth lowers the colonization of *Lactobacillus* in the spleen and intestine, identifying these factors is important for future studies - especially those that implement *Lactobacillus* species as probiotic interventions. Other bacteria common to the intestinal tract, the common opportunistic pathogens *Escherichia-Shigella* and *Staphylococcus*, were also cultured from spleens. However, only one sequence from *Staphylococcus* matched exactly with an ASV from our fecal sequencing data, and no matches were found for *Escherichia-Shigella*. This implies that there could be additional sources of these splenic bacteria other than the intestine, which should be addressed in future studies.

Additional inflammation-related abnormalities experienced by cancer patients, namely splenomegaly and weight loss, were observed in this study. Splenomegaly (often caused by bacterial infection and/or increased proliferation of erythrocyte progenitors) is common in patients with cancer [49]. Splenomegaly was evident in tumor-bearing animals, but was reversed by tumor resection. In these animals, splenomegaly was accompanied by higher bacterial burden and higher levels of the pro-inflammatory cytokines IL-6, CXCL1, and MCP-1. Interestingly, the spleens of these mice exhibited lower levels of TNFα, a pro-inflammatory cytokine that orchestrates diverse anti-microbial responses [50]. This suppression of TNFα may be important for the persistence and viability of increased splenic bacteria, although this also requires further investigation.

Patients with breast cancer often lose weight prior to tumor resection, although weight fluctuations can vary based on staging of the disease [51]. Weight loss in these patients is attributed to high metabolic rate, reduced energy efficiency, and inflammatory signaling elicited by the tumor [52]. However, changes in the intestinal microbiome can also reduce body mass by inducing nutrient malabsorption and intestinal barrier dysfunction [53]. In the present study, tumor-bearing animals experienced weight loss (irrespective of food intake), elevated circulating LBP, and elevated splenic cytokines that were subsequently corrected by tumor resection. Conversely, neither intestinal barrier dysfunction nor fecal bacteriome composition were returned to normal by resection of the tumor. This suggests that numerous factors could influence weight loss in tumor-bearing animals, but that removal of the tumor is sufficient to restore total body mass in resected animals despite persistent changes in intestinal barrier function and the fecal bacteriome. Future studies should determine which of these factors is primarily responsible for weight loss in tumor-bearing animals, and if body composition (and not just total body mass) is restored following tumor resection.

Finally, a unique tumor microbiome has been identified in a multitude of human cancer types, including mammary tumors [22]. However, we were unable to identify detectable amounts of bacteria in the tumors of these mice at sacrifice. This is not surprising given that tumors in this model are grown in vitro and then implanted orthotopically for only a few weeks. This suggests that factors influencing endogenous tumor development may also drive development of the tumor microbiome, which warrants intensive study.

This study has several notable limitations that should be considered in future investigations. We only studied a single tumor type (mammary) in female animals. Other tumor types are likely to alter the microbiome in different ways and therefore may not be permissive of bacterial translocation and systemic inflammation, as well as producing sexually dimorphic effects. These effects may also be dependent on composition of the enteric microbiota, which can be elucidated using gnotobiotic animal models. While we did investigate potential long-term consequences of mammary tumors at 3 weeks post-resection, persistent or permanent alterations in host-microbiota interactions (and thus persistence of GI and behavioral symptoms) remains an open question.

## Conclusions

Overall, we provide evidence that mammary tumors alter the fecal bacteriome and reduce intestinal barrier function, permitting increased systemic enteric bacterial translocation and altered cytokine signaling. These findings provide insight into how tumor biology induces GI and behavioral symptoms and identifies novel host and microbial targets for future therapeutic strategies, such as probiotic *Lactobacillus* supplementation.

## Supplementary Information

**Additional file 1.**

## Data Availability

The datasets used and/or analyzed during the current study are available from the corresponding author on reasonable request.

## Abbreviations

GI: Gastrointestinal
LBP: Lipopolysaccharide Binding Protein
ASV: Amplicon Sequence Variants
