# Supplementary material for: Mammary tumors alter the fecal bacteriome and permit enteric bacterial translocation
Source: BMC Cancer. 2022 Mar 5;22:245. doi: 10.1186/s12885-022-09274-0 (PMC8897840; doi:10.1186/s12885-022-09274-0)
Supplement: Supplementary file 1 — Additional file 1. [file 12885_2022_9274_MOESM1_ESM.docx]

**Supplementary Table 1**

**
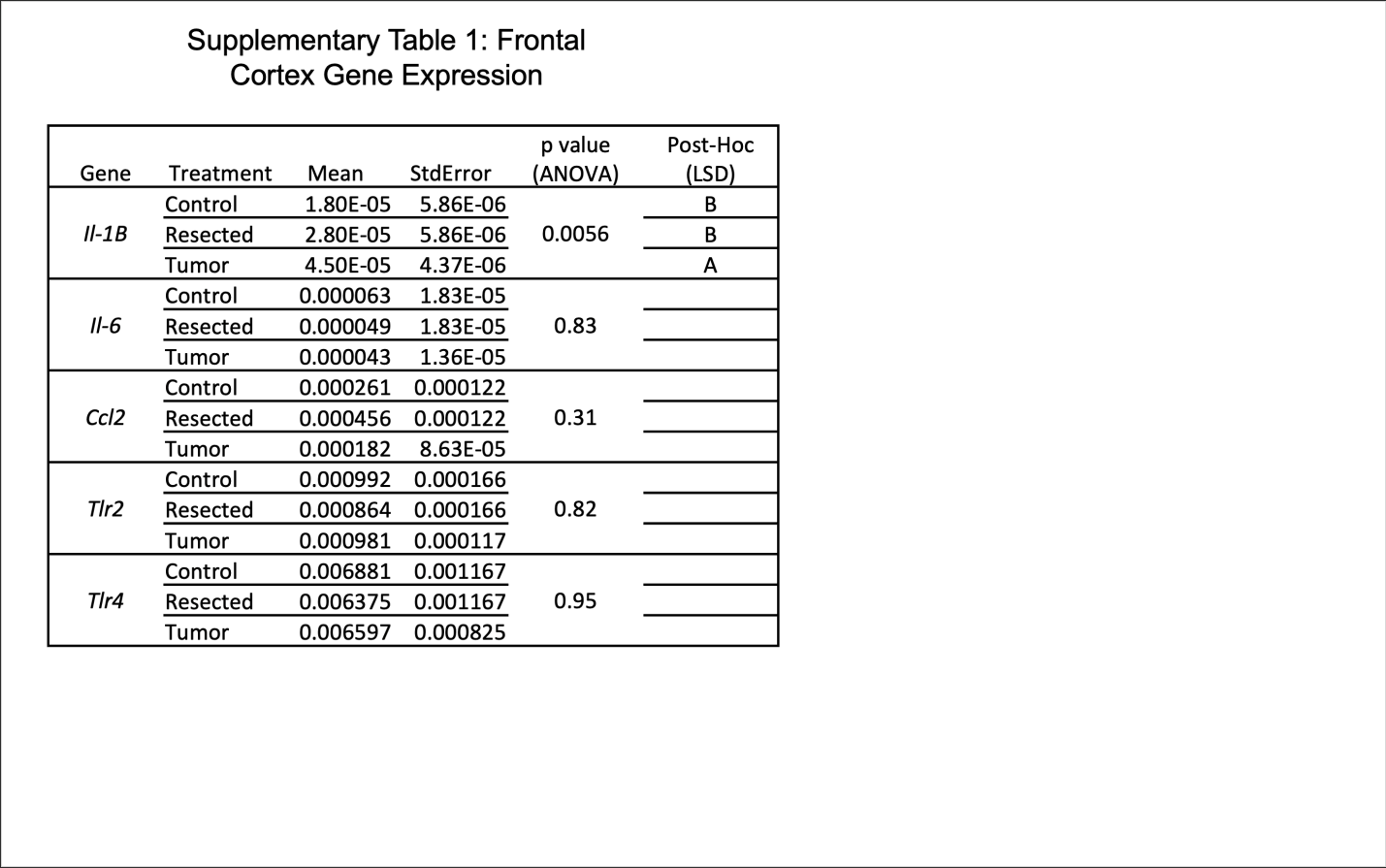
**

**Supplementary Table 1: Brain inflammation-related gene expression.** Among several pro-inflammatory genes in bacterial signaling pathways, only expression of *Il-1B* is higher in the frontal cortex of Tumor-bearing mice and is attenuated by Resection.

**Supplementary Table 2**


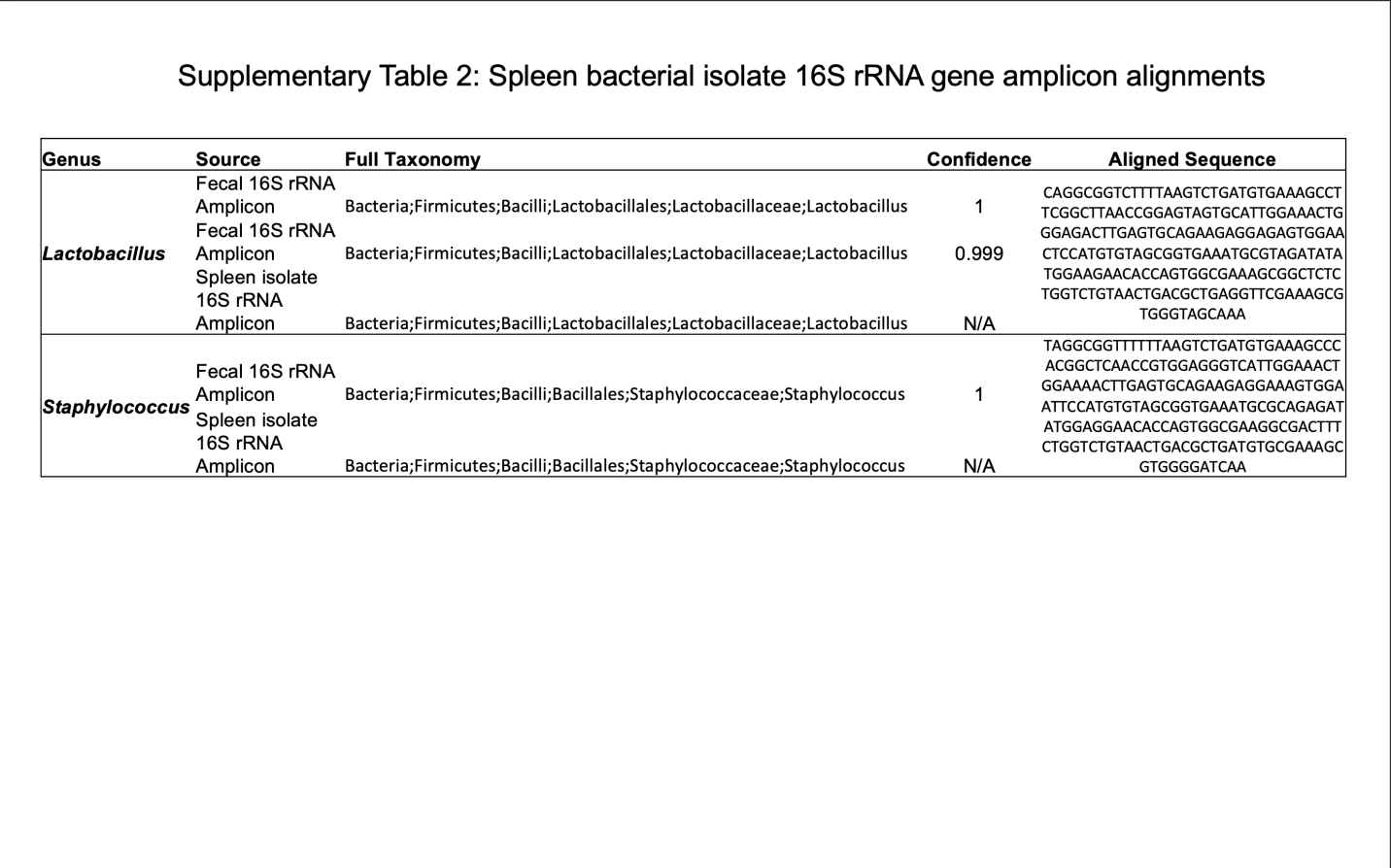


**Supplementary Table 2: Spleen bacterial isolate 16S rRNA gene amplicon alignments.** Isolates from mouse spleens in the genera *Lactobacillus*  and *Streptococcus*  have 100% sequence matches to fecal 16S rRNA amplicon sequences, suggesting that these bacterial translocated from the intestine to the spleen.


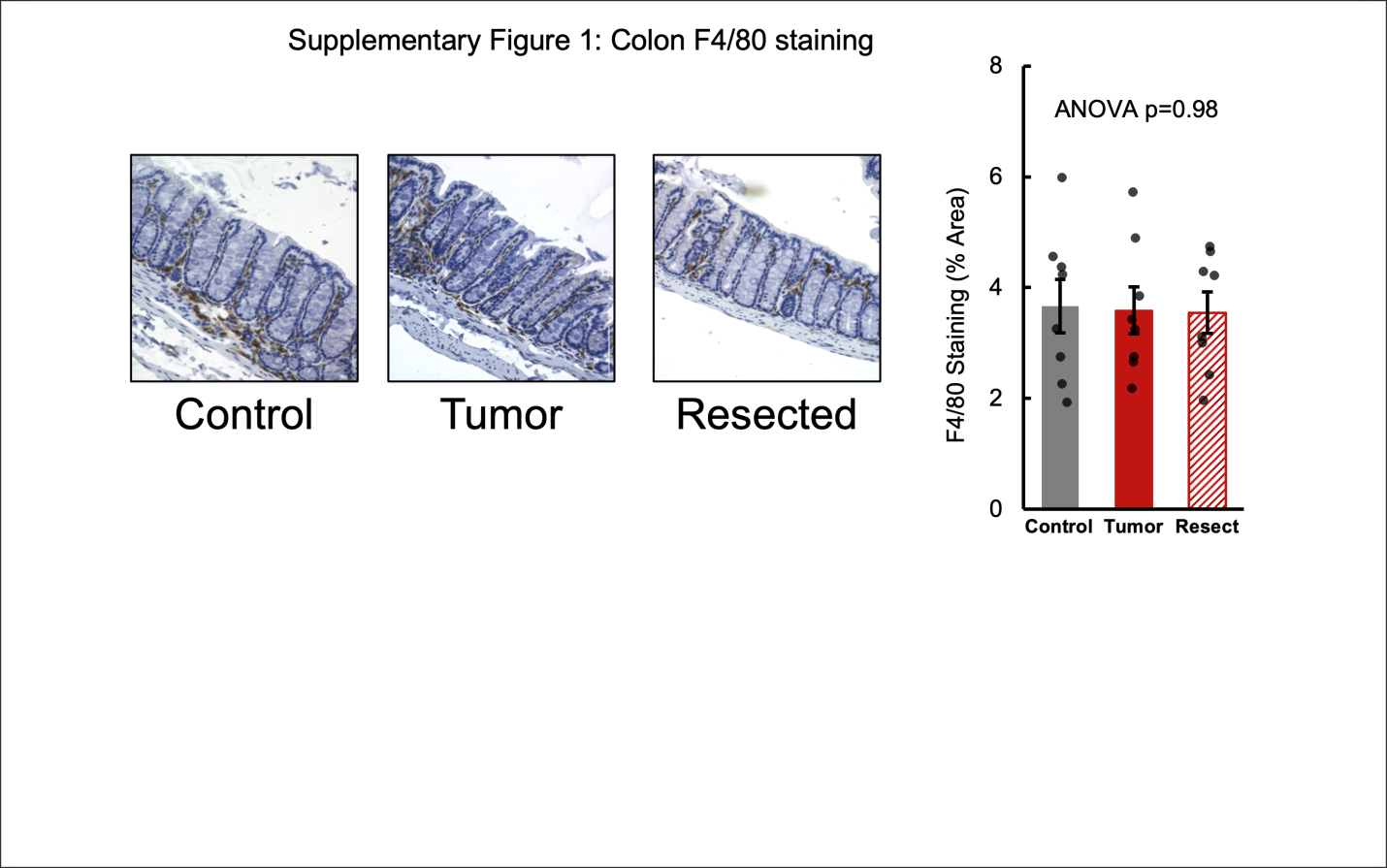


**Supplementary Figure 1: Mammary tumors do not affect colonic monocyte and macrophage infiltration.** Although markers of colonic barrier function are altered by tumor implantation, there is no difference in colonic F4/80 staining between treatment groups, a marker of monocyte and macrophage infiltration.


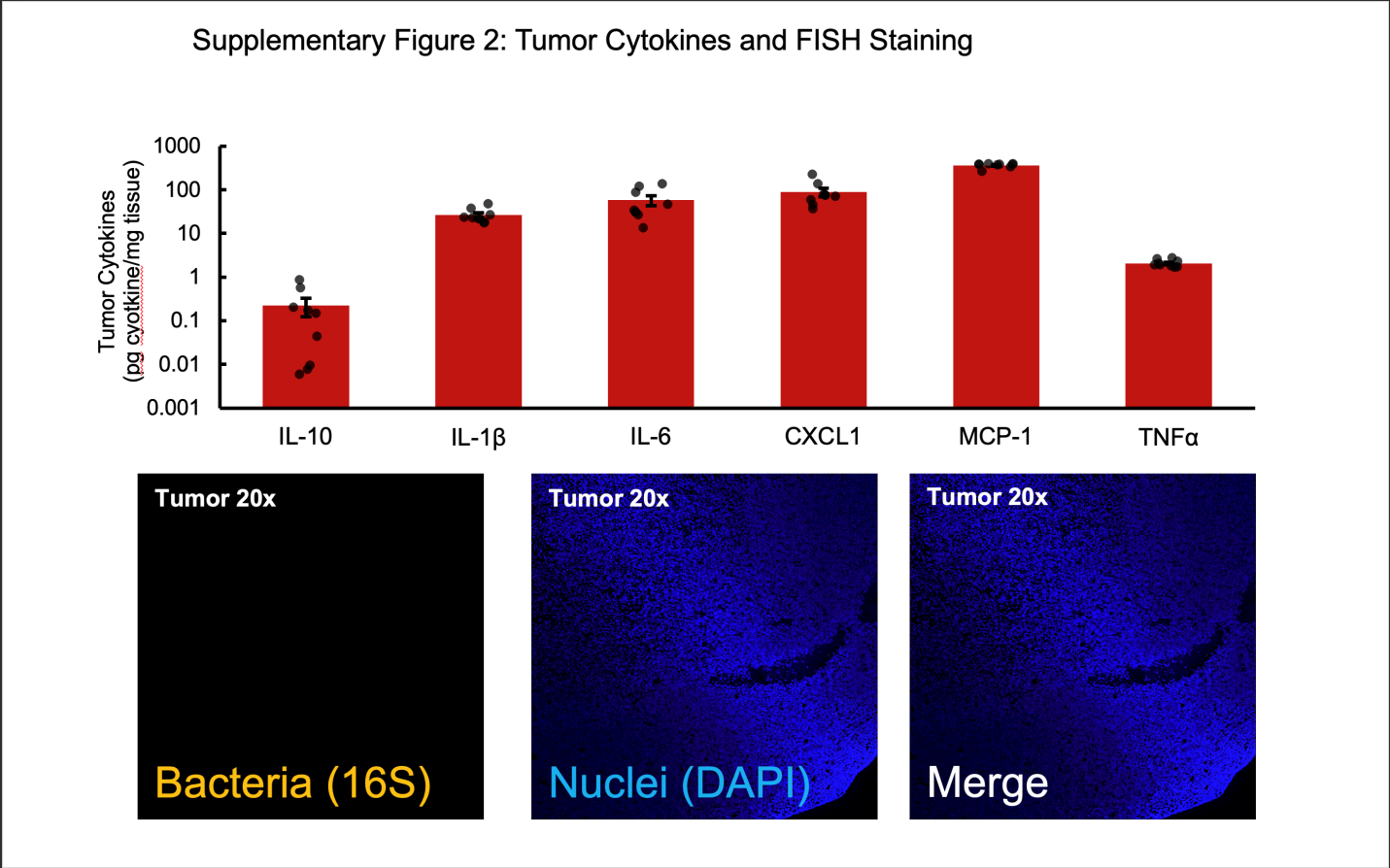


**Supplementary Figure 2: Tumor Cytokines and FISH.** Implanted tumors produce many cytokines, but do not have detectable fluorescence for bacterial DNA.
